# Isolation, Characterization, and Evaluation of Native Rhizobacterial Consortia Developed From the Rhizosphere of Rice Grown in Organic State Sikkim, India, and Their Effect on Plant Growth

**DOI:** 10.3389/fmicb.2021.713660

**Published:** 2021-09-06

**Authors:** Mingma Thundu Sherpa, Laxuman Sharma, Niladri Bag, Sayak Das

**Affiliations:** ^1^Department of Horticulture, School of Life Sciences, Sikkim University, Gangtok, India; ^2^Department of Microbiology, School of Life Sciences, Sikkim University, Gangtok, India

**Keywords:** consortia, bio-control, rhizobacteria, bio-fertilizer, organic agriculture, Sikkim, plant growth promoting rhizobacteria

## Abstract

Eight rhizospheric bacteria were isolated from the organic paddy fields of Sikkim, India, and identified as *Pseudomonas kribbensis* KSB, *Burkholderia cenocepacia* SRD, *Kosakonia oryzendophytica* YMA7, *Pseudomonas rhodesiae* SRB, *Bacillus* sp. ARA, *Paenibacillus polymyxa* COW3, *Bacillus aryabhattai* PSB2, and *Bacillus megaterium* PSB1. They showed plant growth-promoting attributes in rice and have bio-control potential against phytopathogen *Colletotrichum gloeosporioides* of large cardamom (*Amomum subulatum*). *Burkholderia cenocepacia* SRD showed production of indole acetic acid and ammonia and solubilization of phosphate and potassium and also possessed nitrogen fixation potential. It showed antagonistic activity against two other plant pathogens of large cardamom, viz., *Curvularia eragrostidis* and *Pestalotiopsis* sp., under *in vitro* conditions. The liquid bacterial consortium was prepared using the bacterial strains SRB, PSB1, and COW3 (Consortia-1); PSB2, SRD, and COW3 (Consortia-2); and COW3, KSB, and YMA7 (Consortia-3) to increase the growth and yield of rice plants under organic farming conditions. Greenhouse and field studies showed that the Consortia-3 had the highest plant growth-promoting activity. Consortia-3 demonstrated better agronomic performance in terms of root length (9.5 cm),number of leaflets per plant (5.3), grains per panicle (110.6), test grain weight (27.4 g), dry root weight per plant (0.73 g), and total dry biomass per plant (8.26 g).

## Introduction

Northeast India comprises seven sister states, i.e., Assam, Manipur, Meghalaya, Tripura, Mizoram, Arunachal Pradesh, and Nagaland and one brother state, Sikkim. These regions are globally acknowledged for their highest rice diversity (Roy Choudhury et al., 2014). Rice (*Oryza sativa* L.) is one of the main staple food grains of Sikkim that is cultivated in 11,600 ha with a total production of 20,260 tonnes and a productivity of 1.84 t ha^–1^ (Kapoor et al., 2017). It is also described with an epithet “Denzong Valley,” which transcribes to “valley of rice.” Rice is grown during the Kharif season, i.e., monsoon period from July to October. Sikkim alone has greater genetic rice diversity accounting for more than 57 rice accessions documented to date (Kapoor et al., 2017). Among the predominant local rice cultivars, *Attay* is the most common type found all over Sikkim. Depending on the grain size, it can be classified as “*Thulo attay*,” having larger grain size, and “*Sanu attay*,” having small grain size.

Sikkim, the Himalayan state of India, is situated at the 27°N–28°N latitude and 88°E89°E longitude with an elevation ranging from 300 to 6,000 m above the mean sea level (Sherpa et al., 2015; Najar et al., 2018). The state completely banned the application of synthetic fertilizers and pesticides from 2003 and ultimately attained the certified organic status in 2016. Nutrient management in organic farming has attracted the attention of many researchers for exploring the soil microbes as potent bio-fertilizer that can be used either as a single inoculum or as consortia. Numerous researchers have reported the importance of soil bacteria for the production of plant hormones like indole-3-acetic acid (IAA), gibberellic acid (GA3), solubilization of phosphate, potassium, and nitrogen fixation. The most predominant and economically important soil bacteria isolated from agricultural farmlands are *Burkholderia*, *Delftia*, *Pseudomonas*, *Agrobacterium*, *Azospirillum*, *Azotobacter*, *Rhizobium*, *Clostridium*, and *Serratia* (Bhattacharyya and Jha, 2012; Hao and Chen, 2017; Rajawat et al., 2019; Venieraki et al., 2020). Three species from the genera *Bacillus*, viz., *Bacillus luciferensis* K2, *Bacillus amyloliquefaciens* K12, and *Bacillus subtilis* BioCWB, were isolated from the soils of Sikkim and developed as consortia for use in rice and vegetable cultivation for enhancing their nutrient quality and controlling the pest management of the crops (Panneerselvam et al., 2019, 2020).

*Bacillus* species such as *Bacillus thuringiensis*, *Bacillus megaterium*, *B. subtilis*, and *B. amyloliquefaciens* have also been reported for their effectiveness to suppress diseases and pests in plants. Among their different modes of antagonism, antimicrobial peptides (AMPs) such as *bacillomycin*, *iturin*, *surfactin*, and *fengycin* produced by *Bacillus* spp. have been identified and demonstrated to play an important role in suppressing several plant pathogens. Bacteria are also known to produce volatile compounds and soluble metabolites, which play a key role in plant growth and development, stress tolerance, and disease suppression (Panneerselvam et al., 2019). Sikkim has an entirely organic farming system (Kumar J. et al., 2018), and several management practices including indigenous technologies are available for improving plant growth. However, the application of bacterial consortium particularly of native strain to address the nutrient and pest management has been proved to be holistic and ecologically sustainable strategy for agricultural production (Panneerselvam et al., 2020).

Bacterial consortia were developed from the eight native strains isolated from the rice rhizosphere of the organic farming fields of Sikkim, India. Few previous reports were based on either the monocultures or consortia of bacterial strains from the same genera such as *Bacillus* sp. showing the plant growth-promoting (PGP) activity (Panneerselvam et al., 2019, 2020). Our consortia were constituted with isolates from different genera having antifungal properties and good nitrogen, potassium, and phosphorous (NPK) performance and had shown promising PGP activity in both tested greenhouse experiments and field study. The consortia developed was tested in local cultivar *Sanu attay*, for various agronomic performance in terms of root length, the number of leaflets per plant, grains per panicle, test grain weight, dry root weight per plant, and total dry biomass per plant, in the test fields at Pakyong organic farming. The present study attempts to identify some of the novel crop-specific multi-potential PGP bacteria from native rice rhizospheric soils.

## Materials and Methods

### Sampling Sites

**FIGURE 1:**
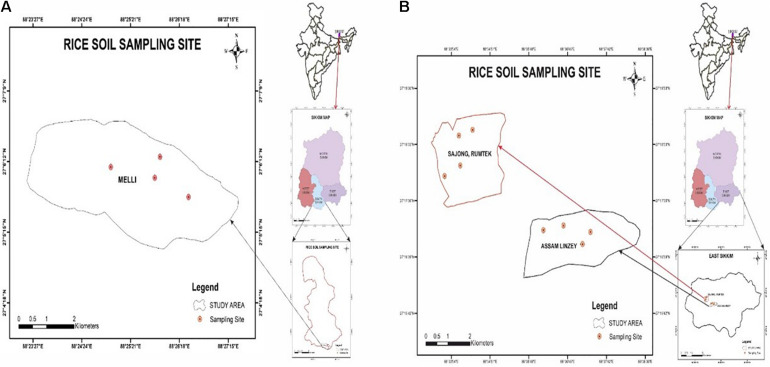
Location of the sampling sites **(A)** South and **(B)** East Sikkim, India.

The geographical location of Melli, Sajong, and Assam Lingzey rice fields was determined by GPSMAP 78S (Garmin, Lenexa, KS, United States) as per the manufacturer’s guidelines. The study areas were the organic rice fields of the progressive farmers from South Sikkim (Melli, and East Sikkim (Sajong and Assam Lingzey) districts of Sikkim, India. Melli (27°06′06.32N; 88°25′38.45E), Sajong (27°18′11.13N; 88°34′26.58E), and Assam Lingzey (27°16′55.98N; 88°37′06.70E) are located at an elevation of 991, 1,268, and 1289 m above the mean sea level, respectively (Figures 1A,B). Four different sampling sites were chosen for the collection of the rhizosphere soil samples from each of these three villages, i.e., Melli, Sajong, and Assam Lingzey.

The cultivation of the crop was done by the farmers in a well-managed contour terrace on hilly and mountainous topography with ridges almost <30% slope. Melli is characterized by a humid subtropical climate with an annual average rainfall of about 3,137 mm and an average temperature of 23°C. Similarly, Sajong and Assam Lingzey are characterized by subtemperate climates with an average rainfall of about 2,578 mm and an average temperature of 16°C. At all the places, the soil was loamy sand; and crops were rain-fed; an assured irrigation source (Bana et al., 2018) was also available.

### Collection of Rhizosphere Soil Samples and Its Physicochemical Analysis

Soil samples were randomly collected from the four different sampling sites at Melli, Sajong, and Assam Lingzey rice fields, during the rainy season of 2019. These samples were collected in triplicates from each of the sampling sites. The field trial was laid out in a split-plot design with local rice cultivar “*Sanu attay*,” a long duration (120 days) variety of paddy. The top 0- to 15-cm soils contained high organic carbon (1%–1.3%) and were slightly acidic in pH (6.5–6.8). One whole paddy plant, after chopping off the shoots, was carefully uprooted (along with the adhering soil; without breaking the secondary and tertiary roots), placed in a polythene bag, labeled and tied (in order to minimize the evaporation loss), and further placed in a box containing ice. The approximate distance of soil adhered to the rice root surface was 12–15 cm. The ice box was transported to a lab where the roots were shaken to dislodge and separate loosely adhering soil aggregates around primary, secondary, and tertiary roots, and the adhering soils were collected and stored in a refrigerator at 4°C for further studies.

These four soil samples collected from the different sampling sites of a village were pooled together and were used for determination of the physicochemical analysis of the soil for that study area. At all the sampling sites, soils are deep, well-drained, fine-loamy soils with loamy surface, having slight stoniness and moderate erosion. They show a slight degree of profile development and are classified as Cumulic Haplumbre and Pachic Haplumbrepts. They occur in association with moderately deep, coarse soils with loamy surface having slight stoniness and moderate erosion. Associated soils are classified as Typic Udorthents and Typic Haplumbrepts. Most of the area is under paddy cultivation; limited extent is under temperate forest (ENVIS, 2007).

Physicochemical parameters such as soil organic carbon (SOC), nitrogen (N), phosphorus (P), potassium (K), and pH of the rice field soil (before and after application of consortia) were analyzed. SOC and available N/P/K of the soil samples were estimated by the ammonium acetate method (Zhang et al., 2019); and the pH of the soil sample was measured by digital pH meter (Mettler-Toledo, India). The soil:water ratio during the sample collection was 1:2. The soil samples were of loamy sand texture. The pH of the Melli and Sajong soil was between 6.7–6.8 and 6.6–6.8, while soil sample pH of Assam Lingzey was recorded as the lowest among other sites, with a pH of 6.4–6.5. The SOC, available nitrogen, phosphorous, and potassium were measured as 1.1%, 238 kg ha^–1^, 19 kg ha^–1^, and 25 kg ha^–1^, respectively, in Melli soil; those of the soil sample of Sajong were recorded as 1.3%, 235 kg ha^–1^, 18.1 kg ha^–1^, and 22 kg ha^–1^, respectively; and those of Assam Lingzey soil sample were measured as 1%, 239 kg ha^–1^, 16.1 kg ha^–1^, and 21 kg ha^–1^, respectively (Supplementary Table 1).

### Isolation and Screening of Plant Growth-Promoting Rhizobacterial Strains, Their Morphological and Biochemical Characterization, and Molecular Identification

Ten grams of rhizosphere soil from each sampling sites was separately suspended in 90 ml of physiological saline (0.85% of NaCl) in a flask and placed on an orbital shaker (at 100 rpm) at 30°C ± 2°C for 1 h. At the end of shaking, the soil samples were serially diluted up to 10^6^ dilutions with physiological saline. Dilutions 10^4^-10^6^ were plated on Pikovskayas’ agar (PA), Aleksandrov’s agar (AA), and Jensen’s agar (JA) as described by Panneerselvam et al. (2019, 2020) by spread plate technique and incubated at 30°C ± 2°C for 48 h. The most prominent colonies were isolated and streaked on PA, AA, and JA plates for obtaining pure culture isolates and further were preserved as in glycerol stock stored at –80°C for further studies.

Colony morphology of the pure bacterial isolates was examined. Gram staining was done as per the universal standard method. The physiological characteristics such as the effect of varying temperature, pH, and NaCl concentrations on the isolates were measured by a UV–Vis spectrophotometer. The optimal temperature for growth was examined by incubating the isolates in various temperatures ranging from 5 to 40°C in nutrient broth. The effect of NaCl concentrations was tested in a range of 1%–5% and pH tolerance in the pH ranging from 4 to 10 in nutrient broth at 30 ± 2°C for 48 h (Arya et al., 2015). The biochemical characterization of the isolates was done by qualitative analysis of various enzymes such as indole, methyl red, Voges–Proskauer, and citrate utilization. The carbohydrate assimilation test was performed using glucose, adonitol, arabinose, lactose, sorbitol, mannitol, rhamnose, and sucrose (Najar et al., 2018).

The bacterial genomic DNA was extracted with the help of HiPurA^TM^ kit (HiMedia, Mumbai, India) as per the manufacturer’s instructions. After extraction of genomic DNA, it was stored at –80°C for further studies. The 16S rRNA genes were polymerase chain reaction (PCR) amplified by using two universal bacterial primers 27F (5′-AGAGTTTGATCCTGGCTCAG-3′) and 1492R (5′-CGGTTAC CTTGTTACGACTT-3′) (Kumar J. et al., 2018). The amplification was done in 50 μl using 4 μl of each dNTP, 2 μl of MgCl_2_, 2 μl of template DNA, 1 μl of each primer (forward and reverse), 1 μl of Taq DNA polymerase, and 33 μl of nuclease-free water (HiMedia, India). Reactions were performed in the Mastercycler gradient (Eppendorf, Chennai, India) with the following reaction conditions; 94°C for 5 min for initial denaturation followed by 30 cycles of 94°C for 30 s, 55°C for 1 min, 72°C for 1 min, and the final extension at 72°C for 10 min (thermal cycler PCR system, BIO-RAD C1000; Bio-Rad Laboratories, Singapore) (Najar et al., 2018). The PCR products were purified with the HiPurA^TM^ PCR clean up system kit (HiMedia, India) and sequenced by ABI Applied Biosystems^TM^ 3500 DNA Analyzer using each universal primer, i.e., 27F and 1492R (Sherpa et al., 2018). The sequences were assembled and aligned with the aid of Codon-Code Aligner software. The sequences were identified using the nucleotide blast tool [National Center for Biotechnology Information (NCBI) search tool)], and the phylogenetic tree was created by using the neighbor-joining method with the Jukes–Cantor evolutionary distance measurement using MEGA v.10 (Saitou and Nei, 1987; Erickson, 2010). After the 16S rRNA gene sequences were obtained, they were matched with the GenBank database using the NCBI Basic Local Alignment Search Tool (BLAST). Identified sequences were submitted to NCBI GenBank data, and accession numbers of the selected isolates were obtained.

### *In vitro* Bioassay for Plant Growth-Promoting Traits

#### Solubilization of Insoluble Phosphate and Potassium

The isolates that were screened for their phosphate-solubilizing ability on PA were streaked and incubated for 72 h at 30°C ± 2°C. The presence of halo zone around the bacterial colony indicated positive isolates. These phosphate solubilization potential isolates were quantitatively estimated in Pikovskayas’ medium enriched with tri-calcium phosphate as an insoluble phosphate source (Panneerselvam et al., 2019). Each of the pure isolated bacterial suspension (0.5 ml of 10^8^ CFU ml^–1^) was inoculated in a 250-ml flask containing 100 ml of Pikovskayas’ broth. After incubation at 150 rpm at 30°C for 7 days in an incubator, the cultures were centrifuged at 1,000 rpm for 25 min. The supernatant was used to measure the soluble P content colorimetrically as described by Ames (1966). Uninoculated flasks containing the same volume of the medium were established as the controls. The solubilized P content was estimated by subtracting the control P from the final P concentration.

The isolates that were screened for their potassium-solubilizing ability on AA were streaked and incubated for 72 h at 30°C ± 2°C. The presence of halo zone around the bacterial colony indicated positive isolates. These potassium solubilization potential isolates were quantitatively estimated in Aleksandrov’s medium (Zhang and Kong, 2014; Paul and Sinha, 2017). For the quantitative estimation of potassium solubilization (Sun et al., 2020), cultures were grown in Aleksandrov’s broth and incubated for 5 days at 30°C in an incubator. After incubation, 5 ml broth was centrifuged at 10,000 rpm for 15 min; and the supernatant was collected and added to 5 ml of sodium cobalt nitrite solution and was incubated at 30°C for 40 min. It was then centrifuged at 10,000 rpm for 10 min. Optical density was taken at 600 nm in a UV–Vis spectrophotometer. Concentration of potassium produced by cultures was measured with the help of standard graph of KCl obtained in the range of 100–1,000 μg ml^–1^.

#### Qualitative Estimation of Siderophore Production

The production of bacterial siderophores was qualitatively estimated by the method as per Schwyn and Neilands (1987). Bacteria were streaked on chrome azurol S (CAS) agar media and incubated at 30°C ± 2°C for 48 h. When the bacteria consumed iron, present in the blue-colored CAS media, orange halos were produced around the colonies, which indicated the presence of siderophores.

#### Production of Indole-3-Acetic Acid

Bacterial isolates were grown in nutrient broth supplemented with 0.5% (w/v) tryptophan (i.e., precursor of IAA) and were compared with broths without tryptophan (control) and incubated at 30°C ± 2°C for 24 h with constant shaking at 150 rpm. The nutrient broth culture was centrifuged at 3,000 rpm for 20 min; and the supernatant was collected in a fresh sterile tube. In a sterile tube, 1 ml of the supernatant was mixed with 2 ml of Salkowski’s reagent (2% 0.5 FeCl_3_ in 35% perchloric acid solution) and kept in the dark. The absorbance [optical density (OD)] was recorded at 530 nm using a UV–Vis spectrophotometer (Lambda PerkinElmer, Waltham, MA, United States). The amount of produced IAA was measured through a standard curve established by commercially procured IAA (0–100 μg ml^–1^) as standard.

#### Qualitative Analysis of Nitrogen-Fixing Ability

The qualitative nitrogen-fixing ability of the bacterial isolates was evaluated based on their ability to grow on N-free Jensen’s media by culturing and incubating them at 30°C ± 2°C for 48 h (Jimtha et al., 2014; Kumar S. et al., 2018).

#### Qualitative and Quantitative Estimation of Ammonia

All the bacterial isolates were qualitatively tested for ammonia production as per Cappuccino and Sherman (1992). The quantitative estimation of ammonia production was assessed by using nutrient broth at 30°C ± 2°C for 24 h with constant shaking at 150 rpm. Cell-free supernatants of nutrient broth were added with 5% Nessler’s reagent, and uninoculated nutrient broth with Nessler’s reagent served as a control. Color changes of supernatant from pale to deep yellow were observed for positive isolates. Absorbance was measured at 425 nm, and the amount of ammonia produced was estimated using the ammonium sulfate standard curve of concentrations in (0–100 mM) range (Chrouqi et al., 2017).

### Assessment of the *in vitro* Antifungal Activity of Bacterial Isolates

The antifungal activity of the bacterial isolates was evaluated against the fungal pathogens infecting large cardamom (*Amomum subulatum*) of Sikkim by dual culture assay using Potato Carrot Agar (PCA). The following large cardamom fungal pathogens were provided by the Department of Horticulture, Sikkim University, Sikkim, India, viz., *Colletotrichum gloeosporioides* 05 (MN710587), *Curvularia eragrostidis*04 (MN710527), and *Pestalotiopsis* sp. 02 (MN710582) used for the assay. Agar disc (5 mm) of phytopathogens for 5-day-old culture was placed at one pole of the Petri’s plate, and 24-h-old bacterial culture was streaked on the opposite pole (Panneerselvam et al., 2019). Antifungal activity of the bacterial strains was determined by comparing with the control plates inoculated with the fungus only. Inhibition of fungal mycelium (halo zone) around the bacterial colony was a criterion for positive reaction, and its zone of inhibition was measured. The fungal growth was monitored at 30°C ± 2°C for 120 h; and the three replications per isolate were considered. Fungal colony diameter (growth) was measured, and the percentage of inhibition was calculated as per the methods suggested by Lahlali and Hijri (2010).

$$P\,e\,r\,c\,e\,n\,t\,a\,g\,e\,o\,f\,i\,n\,h\,i\,b\,i\,t\,i\,o\,n=\left(C_{d}-\frac{T_{d}}{C_{d}}\right)\times 100$$

where *C*^*d*^ is the colony diameter (mm) of the control and *T*^*d*^ is the colony diameter (mm) of the test plate Antagonism was also assessed under potato dextrose broth methods wherein first the mycelia dry weight was calculated from which the percentage inhibition by bacteria was calculated as per the formula described by Lahlali and Hijri (2010).

$$P\,e\,r\,c\,e\,n\,t\,a\,g\,e\,o\,f\,i\,n\,h\,i\,b\,i\,t\,i\,o\,n=\left(C^{w}-\frac{T^{w}}{C^{w}}\right)\times 100$$

where *C*^*w*^ mycelia weight (g) is in the control and *T*^*w*^ mycelia weight (g) is in the treatment broth.

### *In vitro* Bacterial Compatibility Test

Only the selected bacterial strains were investigated for their compatibility as described by Raja et al. (2006). Each pure bacterial isolate was cultured individually in Luria Bertani broth at 30°C ± 2°C in a shaker cum incubator at 100 rpm for 48 h. Later on, all the strains were cross-streaked on Luria Bertani agar plate. The cross-streaked plates were incubated at 30°C ± 2°C for 48 h and then examined for the formation of inhibition zones around the colonies.

### Preparation of Bacterial Consortia

The selected isolates SRB, SRD, PSB1, PSB2, COW3, KSB, and YMA7 were grown until the stationary phase (2 × 10^9^ cells ml^–1^). Based on the compatibility test, NPK-producing consortia were prepared such as SRB (K), PSB1 (P), and COW3 (N) (Consortia-1); PSB2 (K), SRD (P), and COW3 (N) (Consortia-2); and COW3 (N), KSB (P), and YMA7 (K) (Consortia-3). The selected individual pure bacterial strains having potassium-solubilizing, phosphorous-solubilizing, and nitrogen-fixing abilities were inoculated into 100-ml conical flask containing each of 50 ml of nutrient broth and was incubated for 48 h at 30°C. The bacterial consortia were prepared by inoculating each of the 200 μl of 48-h-old culture (concentration of 2 × 10^9^ CFU ml^–1^) into 1,000-ml conical flask containing 500 ml of nutrient broth supplemented with 5% sucrose. It was incubated in shaker cum incubator at 150 rpm at 30°C for 48 h. Then the consortia were centrifuged at 4,000 × *g* for 5 min and were washed twice with sterile phosphate-buffered saline PBS (1.24 g of K_2_HPO_4_, 0.39 g of KH_2_PO_4_ and 8.80 g of NaCl per liter). The supernatant was discarded, and the pellet was suspended in PBS buffer. The viable count of the suspension was adjusted by adding sterile distilled water to give a final concentration of 2 × 10^9^ cells ml^–1^ (2 × 10^9^ CFU ml^–1^) with the help of a hemocytometer (Marienfeld, Lauda-Königshofen, Germany).

### *In vivo* Root Colonization and Plant Growth Assessment Through Greenhouse Pot Experiment

The effect of the bacterial consortia on plant growth was examined on rice (local cultivar *Sanu Attay*) in a pot at the greenhouse (Department of Horticulture, Sikkim University) in a randomized complete block design method with three replicates. Rice seeds were surface-sterilized with 95% ethanol for 5 min and washed several times with sterilized distilled water.

Three different bacterial consortia [Consortia-1 (*Pseudomonas rhodesiae* SRB +*B. megaterium* PSB1 +*Paenibacillus polymyxa* COW3), Consortia-2 (*Bacillus aryabhattai* PSB2 +*Burkholderia cenocepacia* SRD +*P. polymyxa* COW3), and Consortia-3 (*P. polymyxa* COW3 +*Pseudomonas kribbensis* KSB +*Kosakonia oryzendophytica* YMA7)] were grown in a nutrient broth supplemented with 5% sucrose and was incubated at 30°C for 48 h in an orbital shaker at 150 rpm. Rice seeds were inoculated with each of the bacterial consortia for 5 h at room temperature before planting in pots. Control seeds were also treated in the same manner with sterilized distilled water.

Each pot contained 3 kg of autoclaved sterile soil. Each of the bacterial consortia inoculated seeds was planted 1 cm below the soil surface in each pot. Three replications were conducted for all the treatments. The pots were irrigated with sterile distilled water every day. Rice roots were harvested at the end of the trial, and their dry weight was measured.

### Determination of N, P, and K Uptake by the Rice Plant Grown in Greenhouse Pot Experiment

The availability of N/P/K uptake by the rice plant grown in greenhouse pot was estimated by the analysis of the soil during each treatment, i.e., at initial stage and after 60 days of treatment. In case of the first treatment, i.e., at the initial stage, the soil samples from 0.45 m depth were randomly collected from the pot for each treatment with the three different bacterial consortia. The soil samples were aseptically collected with the help of screw auger. The samples were brought to the laboratory and air-dried under room conditions for 2 days. To remove the further moisture in the soil, the samples were dried in hot air oven at 35°C ± 2°C for 6 h. Then the dried soil samples were grinded by wooden roller and thereafter manually sieved through 2 mm stainless steel sieve. The fine-powdered samples were then processed for their chemical analysis through tri-acid mixture.

In case of the second treatment, the effect of the bacterial consortia on nutrient uptake of rice plant was analyzed in the 60-day-old plants. The plant samples from the greenhouse pot experiment were brought to the laboratory, the whole rice plant was air-dried for 2–3 days, and after that, it was dried in a hot air oven at 60°C ± 2°C overnight to achieve complete dryness of the samples. Once the plant samples were completely dry, they were grinded to powder form and passed through 2 mm stainless steel sieve manually. The filtered powder was then processed for the various chemical assays through tri-acid mixture.

Total nitrogen (N) was assessed by Kjeldahl digestion method; total phosphorous (P) was evaluated by ammonium-molybdate technique in acid digestion procedures; and potassium (K) was estimated by flame photometric methods (Duarah et al., 2011) for both the soil samples (during initial treatment) and plant samples (during second treatment, i.e., after 60 days’ growth in a greenhouse pot experiment).

### *In vivo* Plant Growth-Promoting Rhizobacteria Activity of the Consortia in Field-Based Trials

The bacterial consortia were applied at the rice field at Pakyong (27°13′45.12 N and 88°35′33.26 E, and elevation is 1,272 m above the mean sea level), East Sikkim, in triplicates. Soils are deep, well-drained, fine-loamy soils with loamy surface, have slight stoniness and moderate erosion, and are classified as Cumulic Haplumbre and Pachic Haplumbrepts. The consortia were applied to the field area of 36.57 m × 60.96 m (2,229.3 m^2^) where local rice variety *Sanu attay* was organically cultivated. The consortia were administered to 25-day-old rice plant saplings through root dipping method (Fasusi et al., 2021). The uninoculated rice saplings were the controls for the study. The PGP traits were observed in the plants after 60 days by transplanting in organic agricultural farming fields.

### Statistical Analysis

Data of bacterial consortia treatments were compared by the least significance difference (LSD) test using R software (Devkota et al., 2019). The differences at the *p* ≤ 0.05 value were considered as significant results.

## Results

### Bacterial Isolation and Biochemical Characterization

A total of 25 PGP bacteria were screened and isolated from the rice rhizospheric soil. Based on the morphological, biochemical characterization, and PGP attributes, eight bacterial isolates were selected for further analyses. The cell morphology of the isolates was Gram-positive and Gram-negative rods. Most of the isolates were Voges–Proskauer negative, methyl red positive, and citrate utilization test positive, i.e., seven isolates, six isolates, and seven isolates. The carbohydrate assimilation test showed that most of the isolates fermented carbohydrates like glucose, arabinose, and sucrose (Supplementary Table 2). The physiological analysis showed that isolates could tolerate a wide range of temperature, pH, and NaCl concentrations. Growth was observed up to 5% NaCl concentration (Supplementary Figures 1A,B). The isolates could actively grow in the temperature range from 10 to 40°C. However, most of the isolates showed optimum growth temperature at 30°C (Supplementary Figures 1C,D). The isolates were able to grow in both acidic and alkaline conditions of pH ranging from 4.0 to 10.0 (Supplementary Figures 1E,F). However, the optimum pH for most of the isolates was pH 8.0, although few isolates showed growth up to pH 10 (SRB, KSB, and YMA7).

### Identification of Bacteria

Molecular identification revealed the singular dominance of the genus *Bacillus*. The other genera found in this study were *Burkholderia*, *Kosakonia*, and *Pseudomonas*. Identified isolates of *Bacillus* were *B. aryabhattai* PSB2 (MW020338), *B. megaterium* PSB1 (MW020222), and *Bacillus* sp. ARA (MW021509). Similarly, identified isolates of *Pseudomonas* were *P. kribbensis* KSB (MW308683) and *P. rhodesiae* SRB (MW020262), while other identified isolates were *Kosakonia oryzendophytica* YMA7 (MW020337), *P. polymyxa* COW3 (MW020264), and *B. cenocepacia* SRD (MW020263). The alignment and similarity search of 16S rRNA sequence with nr/nt database of NCBI have shown that many of the isolates have a percentage of identity >98%. The identified species, the percentage of identity, and their NCBI accession number are given in Table 1. The phylogenetic tree was made with the help of MEGA v.10 software using the maximum likelihood method and the Jukes–Cantor model as shown in Supplementary Figure 2.

**TABLE 1:** Identification of bacteria based on 16S rRNA, the percentage of identity, and NCBI accession numbers.

**Isolates**	**Partial identification based on 16S rRNA gene sequencing**	**% identity**	**Accession no.**
PSB1	*Bacillus megaterium*	98	MW020222
COW3	*Paenibacillus polymyxa*	99	MW020264
SRB	*Pseudomonas rhodesiae*	99	MW020262
ARA	*Bacillus* sp.	99	MW021509
KSB	*Pseudomonas kribbensis*	99	MW308683
YMA7	*Kosakonia oryzendophytica*	99	MW020337
SRD	*Burkholderia cenocepacia*	98	MW020263
PSB2	*Bacillus aryabhattai*	99	MW020338

### Plant Growth-Promoting Activity

In this study, eight efficient isolates were selected based on their PGP traits, in particular, (i) the solubilization of phosphate and potassium; (ii) production of IAA and siderophore; and (iii) ability to fix nitrogen. The selected bacterial isolates were identified as *P*. *rhodesiae* SRB, *B*. *megaterium* PSB1, *P. polymyxa* COW3, *B. aryabhattai* PSB2, *B. cenocepacia* SRD, *Bacillus* sp. ARA, *P. kribbensis* KSB, and *K. oryzendophytica* YMA7 (Table 1). The quantitative estimation of phosphate and potassium indicated that isolate *B. cenocepacia* SRD produced significantly higher phosphate (530 μg ml^–1^) and potassium (581 μg ml^–1^) than did the other isolates (Table 2). Similarly, quantitative estimation of IAA and ammonia showed that the isolate *K. oryzendophytica* YMA7 produced a considerably higher extent of IAA (84 μg ml^–1^) and ammonia (61 mM) than did the other isolates (Table 2). However, out of eight isolates, *P. polymyxa* COW3 and *B. aryabhattai* strain PSB2 had only the nitrogen-fixing ability.

**TABLE 2:** PGP traits of isolated bacterial isolates.

**Strain**	**Phosphate (μgml^–1^)**	**Potassium (μg ml^–1^)**	**IAA (μg ml^–1^)**	**Ammonia (mM)**	**Siderophore production**	**Nitrogen fixation**
PSB1	460 ± 0.25	250 ± 0.071	21.0 ± 6.1	7.0 ± 2.13	++	+
COW3	420 ± 0.005	250 ± 0.00	25.0. ± 0.66	5.0 ± 0.00	+	**+++**
SRB	210 ± 0.003	580 ± 0.008	5.0 ± 0.33	10.0 ± 0.33	+	+
ARA	480 ± 0.03	570 ± 0.26	4.0 ± 0.00	6.0 ± 0.00	+	++
KSB	410 ± 0.003	**590 ± 0.01**	59.0 ± 0.33	35.0 ± 0.33	++	+
YMA7	517 ± 0.01	570 ± 0.03	**84.0 ± 1**	**61.0 ± 1.2**	**+++**	++
SRD	**530 ± 0.008**	581 ± 0.012	20.0 ± 0.66	5.0 ± 0.66	+	++
PSB2	450 ± 0.006	330 ± 0.003	10.0 ± 0.00	2.0 ± 0.33	+	+

*Data are presented as mean of triplicates ± standard deviation. “+” denotes weakly positive; “++” denotes moderately positive; and “+++” denotes strongly positive. Bold values indicate the highest value obtained among all the strains for the specific PGP trait. PGP, plant growth promoting; IAA, indole-3-acetic acid.*

### Antagonistic Activity Against Pathogenic Plant Fungi

The dual-plate studies revealed that *B. cenocepacia* SRD had higher antagonistic activity against rice sheath blight and large cardamom leaf spot disease-causing fungi *C. gloeosporioides* (90%–91%), *C. eragrostidis* (43%–49%), and *Pestalotiopsis* sp. (29%–33%) (Supplementary Table 3 and Supplementary Figure 3). Similarly, *K. oryzendophytica* YMA7 showed 56% antagonism against *C. eragrostidis* and *C. gloeosporioides* (53%) and 27% with *Pestalotiopsis* sp., respectively, in both culture plate and broth assay (Supplementary Tables 3, 4). Furthermore, compatibility assays conducted on Nutrient Agar plate deciphered that all the tested isolates have no antagonistic effect on each other such as Consortia-1 (*P*. *rhodesiae* SRB, *B*. *megaterium* PSB1, and *P. polymyxa* COW3), Consortia-2 (*B. aryabhattai* PSB2, *B. cenocepacia* SRD, and *P. polymyxa* COW3), and Consortia-3 (*P. polymyxa* COW3, *P. kribbensis* KSB, and *K. oryzendophytica* YMA7).

### Root Growth Stimulation Potential

Greenhouse pot assessments of selected bacterial consortia on rice roots growth have shown the development of the rice root system as a function of IAA production. Consortia-3 (*K. oryzendophytica* YMA7 +*P. kribbensis* KSB +*P*. *polymyxa* COW3) stimulated the maximum amount of lateral roots on rice plant as compared with other consortia. The root length of rice exhibited by all the three different bacterial consortia were higher than that of the control.

### Evaluation of Plant Growth-Promoting Traits in Field Study

**FIGURE 2:**
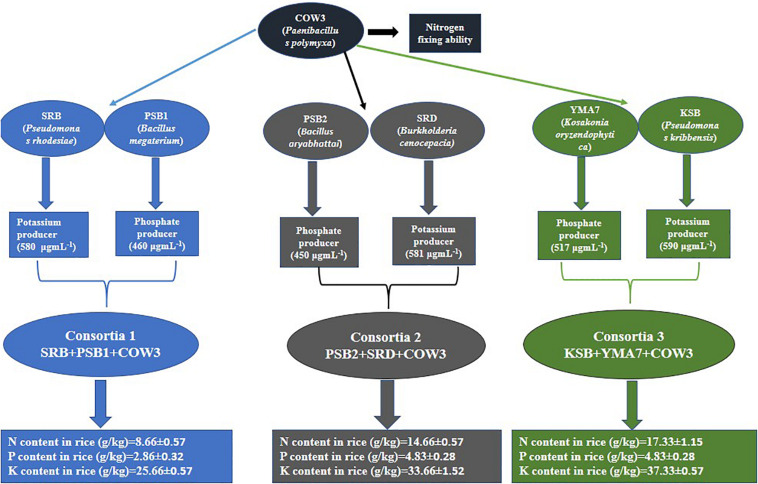
Flowchart representation of the PGP traits as shown by the individual isolates and consortia.

**FIGURE 3:**
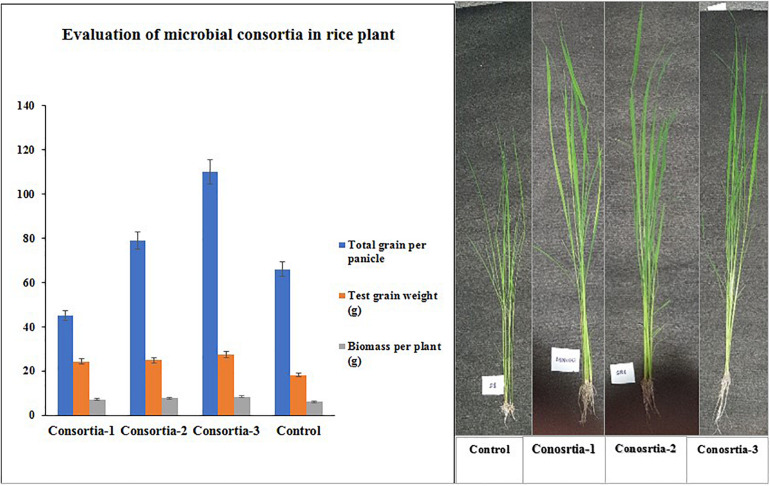
Evaluation of three native consortia (Consortia-1, 2, and 3) on rice plants. Values are means ± SE.

**FIGURE 4:**
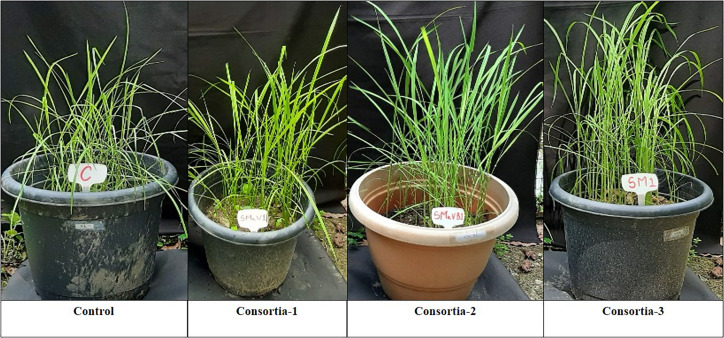
Phenotype differences of potted rice plant under different consortia treatments. Consortia-1 (SRB+PSB1+COW3), Consortia-2 (PSB2+SRD+COW3), and Consortia-3 (COW3+KSB+YMA7), and control rice plant.

Three bacterial consortia developed in this study were first tested in greenhouse pot experiments and later on applied to the rice field (Figure 2). Based on the agronomic parameters, significant increases were observed in all the plant growth and yield parameters except leaf number per plant when compared with uninoculated rice plant (Table 3). All the three bacterial consortia significantly improved grains per panicle (C1:45.0, C2:79.0, and C3:110 grain numbers per panicle) (Figures 3, 4 and Supplementary Figures 4, 5), grain weight in grams (C1:24.3 g, C2:24.8 g, and C3:27.8 g) (Figures 3, 4 and Supplementary Figures 4, 5), and root length in cm (C1:6.6 cm, C2:9 cm, and C3:9.5 cm) as compared with uninoculated control rice plants. However, among the three bacterial consortia, Consortia-3-inoculated rice plants showed significantly higher biomass (8.26 g/plant), grains per panicle (110 grain/panicle), test grain weight (27.4 g), root length (9.5 cm), and dry root weight (0.73 g/plant)as compared with the other consortia (Table 3). The combined PGP traits such as phytohormone production and nutrient solubilization abilities were maximally observed in Consortia-3 (*K. oryzendophytica* YMA7+*P. kribbensis* KSB +*P*. *polymyxa* COW3). The potential assessment of the bacterial consortia application improved the soil N, P, K value as compared with the control; but in our study, interestingly, the pH value of the experimental field soil decreased from 6.5 to 6.0 (Supplementary Tables 5, 6). This result might be due to the fact that the bacterial colonization in soil decreases pH value due to the secretion of organic acid by bacteria as secondary metabolites.

**TABLE 3:** Effect of bacterial consortia inoculation on plant growth promotion after 60 days of transplanting of rice at farmer’s field.

**Microbial consortia**	**Tiller number per bunch (5 plants)**	**Root length (cm)**	**Number of leaflets per plant**	**Grains per panicle**	**1,000-grain wt. (g)**	**Dry root dry wt. per plant (g)**	**Total dry biomass per plant (g)**
C1-Consortia-1 (SRB+PSB+COW3)	14.3 ± 1.45^ab^	6.6 ± 0.66^b^	4.0 ± 0.00^b^	45.0 ± 3.51^c^	24.3 ± 0.88^b^	0.49 ± 0.47^bc^	7.16 ± 0.20^c^
C2-Consortia-2 (PSB2+SRD+COW3)	15.3 ± 0.33^a^	9.0 ± 0.00^a^	5.3 ± 0.33^a^	79.3 ± 6.56^b^	24.8 ± 0.57^b^	0.56 ± 0.41^b^	7.66 ± 0.28^b^
C3-Consortia- (COW3+KSB+YMA7)	12.6 ± 0.88^b^	9.5 ± 0.00^a^	5.3 ± 0.33^a^	110.6 ± 9.17^a^	27.4 ± 0.42^a^	0.73 ± 0.03^a^	8.26 ± 0.25^a^
Uninoculated control	12.3 ± 0.88^b^	3.5 ± 0.28^c^	4.3 ± 0.33^b^	66.6 ± 1.73^b^	18.2 ± 0.34^b^	0.42 ± 0.09^c^	6.03 ± 0.05^d^
LSD (*p* ≤ 0.05)	2.579	1.412	0.998	19.892	1.968	0.12	0.39
CV	9.44	9.86	10.52	13.20	2.55	11.69	2.71

*Values are means ± SE. a,b,c, and d letters on the bars denote differences on the basis of a *t*-test (*p* < 0.05).*

### Determination of N/P/K Content in Rice Plant

**FIGURE 5:**
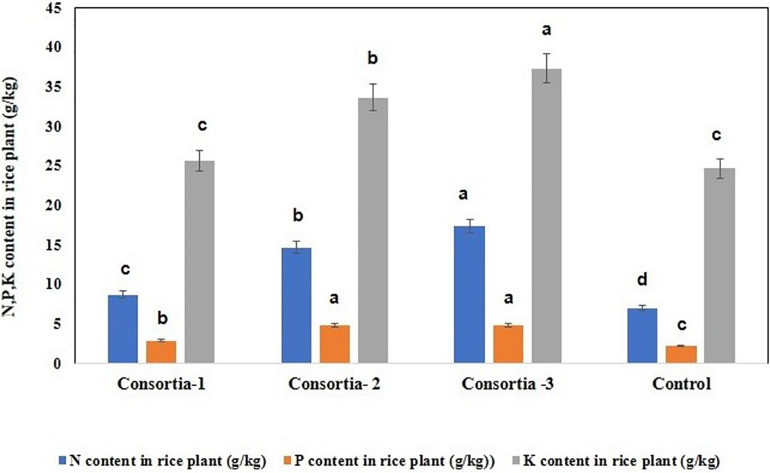
Nitrogen, phosphorus, and potassium uptake for rice plant after consortia application (60 days trail). Values are means ± SE. abcde letters on the bars denote differences on the basis of a *t*-test (*p* < 0.05).

In order to verify whether consortia-based treatment can promote nutrient uptake by rice plants, the content of nitrogen, phosphorus, and potassium in rice plant were determined. Our results showed that significant increase in rice plant N/P/K uptake was observed when the soil was inoculated with different bacterial consortia as compared with the uninoculated control plant. Inrice plant, in the soil inoculated with three different bacterial consortia (Consortia-1, Consortia-2, and Consortia-3), the plant N/P/K content was N (8.66, 14.66, and 17.33 g kg^–1^), P (2.86, 4.83, and 4.83 g kg^–1^), and K (25.66, 33.66, 37.33 g kg^–1^) and control plant N/P/K (7.0, 2.16, and 24.66 g kg^–1^) (Figure 5 and Supplementary Table 7).

## Discussion

An assortment of abiotic and biotic elements shape soil-and plant-related living spaces and adjust the creations and exercises of their microbial networks, which thus bear upon the nature of their development of plants and the creation of root exudates (Jain et al., 2020). Bacteria harbor in roots, depending on the incredible variety of natural root exudates, which in the long run influences the growth and development of the plant (Ngalimat et al., 2021). Here, in this study, we examined the impact of rice rhizosphere regulated with local bacterial consortia developed to increase the uptake the N/P/K as nutrients from the soil.

Bacterial isolation was done from soil rhizosphere fractions by 16S rRNA gene sequencing. This technique offers a culture-independent method for tracking dominant bacterial populations in soil (Sultana et al., 2020). To the best of our knowledge, this study represents the first approach using culture-independent method to design a native consortia that can enhance the rice crop nutrient quality uptake as in N/P/K from the soil of organic farming in Sikkim. In brief, soil samples from three different organic paddy cultivation field sites in Sikkim, India, were chosen [M (loamy sand), S (loamy sand), and AL soil (loamy sand)] to screen and isolate PGP rhizobacteria (PGPR) and design a consortia that can uptake N/P/K nutrients from soil. These bacterial isolates also had antifungal properties that were effective against fungal pathogens. To validate the consortia performance, chemical analysis of soil (before consortia administration and post-consortia administration) was compared in greenhouse pot experiments. Later on, field-based trials for 60 days on application of consortia were also measured to verify the various agronomic parameters.

Antagonistic and PGPR were screened and isolated from the rhizosphere of rice and was identified through a polyphasic approach, based on morphological, biochemical, and partial 16S rRNA gene sequencing. The cultures isolated were four *Bacillus* sp. strains (PSB1, PSB2, COW3, and ARA), two *Pseudomonas* sp. strains (SRB and KSB), and one each strain of *Burkholderia* sp. (SRD) and *Kosakonia* sp. (YMA7).16S rRNA gene sequencing analysis and homology with reference strains from the nucleotide database of NCBI showed that the strains PSB1, PSB3, ARA, COW2, SRB, KSB, SRD, and YMA7 have average nucleotide identity percentage ranges from 98 to 99% with *B. megaterium*, *B. aryabhattai*, *Bacillus* sp., *P. polymyxa*, *P. rhodesiae*, *P. kribbensis*, *B. cenocepacia*, and *K. oryzendophytica*, respectively.

The *B. megaterium* strain PSB1 isolated in this study showed high salt tolerance at 8% NaCl as compared with other isolates.

Many studies revealed that the genus *Pseudomonas* represents the dominance of PGPR for many crops (Qessaoui et al., 2019). In the present investigation, *P. kribbensis* strain KSB showed multiple PGP activities including siderophore production. This result corroborates with previous findings wherein multiple PGP traits have been described from *Pseudomonas* sp. isolated from the rhizospheric soil of wheat, barley, and rice (Ahmad et al., 2008; Sharma et al., 2011).

The member of the genus *Kosakonia* consists of seven different species, *K. oryzendophytica* (Hardoim et al., 2013), *Kosakonia cowanii* (Inoue et al., 2000), *Kosakonia radicincitans* (Kämpfer et al., 2005), *Kosakonia oryzae* (Peng et al., 2009), *Kosakonia arachidis* (Madhaiyan et al., 2010), *Kosakonia sacchari* (Zhu et al., 2013), and *Kosakonia oryziphilus* (Hardoim et al., 2013), which belong to the family Enterobacteriaceae. Except for *K. cowanii*, which is considered to be from clinical origin, other species of the genus are nitrogen-fixing bacteria, which are commonly associated with plants (Li Y. et al., 2017). They are most frequently found in the nitrogen-fixing bacterial community of some non-leguminous plant, such as rice (Hardoim et al., 2013) and sugarcane (Raju et al., 2020). The *Kosakonia* species contains flagella, which enable them to swim and possibly help in the attachment to the plant surface. It might also produce different secretion systems that help to interact with both host plant and associated microbiota (Becker et al., 2018). The present *in vitro* screening for characteristics generally associated with PGP showed that *K.oryzendophytica* strain YMA7 showed a higher production of IAA (84 μg ml^–1^) and ammonia (61 mM). It also showed higher solubilization for potassium (570 μg ml^–1^) and phosphate (517 μg ml^–1^). It also produced siderophores. It also produced a high amount of phosphate (517 μg ml^–1^) as compared with *K. oryzendophytica* strain NRCSSDCU262 (207 μg ml^–1^), which is an endophytic-rhizospheric phosphate-solubilizing bacteria (PSB) isolated from cumin grown in agricultural fields of Rajasthan, India (Devi et al., 2020).

Other most important soil bacteria that belong to the genus *Burkholderia* in the class Betaproteobacteria (Castanheira et al., 2015) have also been reported as one of the dominant extracellular PGPR for many crops (Becker et al., 2018). *Burkholderia* species in general have symbiotic relationship with plants, functioning as active rhizospheric components (Castanheira et al., 2015), endophytic plant colonizers, or microsymbionts in legume root nodules, as reported by many researchers (Sutton, 1992). The ability to fix nitrogen was demonstrated by several *Burkholderia* spp. associated with different plants, for example, maize, coffee (Estrada-de los Santos et al., 2001), sugarcane, and tomato (Lin et al., 2012; Paungfoo-Lonhienne et al., 2014), had already been reported in several studies. In our study, regarding plant growth promotion traits, *B. cenocepacia* SRD showed higher solubilization of phosphate and potassium. It also produced IAA and also showed antagonistic activity against plant fungus *C. gloeosporioides*.

*Colletotrichum* is a broad-spectrum plant pathogen infecting a host range of plants. Its pathogenicity leads to major losses of crops and other agricultural products (Saju et al., 2013). They play a significant role in causing post-harvest loss (Sutton, 1992). The disease symptoms vary on plant species; but generally, it has been observed to affect plant leaves, stems, and fruits of the host plant (Panneerselvam et al., 2019). The antagonistic PGP microbes directly compete with the plant pathogens for nutrition and inhibit or reduce the pathogen growth via hyper-parasitism (Sutton, 1992). Our dual-plate studies on *B. cenocepacia* SRD showed 90%–91% antagonistic activity against *C. gloeosporioides*, which is a rice sheath blight and large cardamom leaf spot disease-causing fungi predominantly found in Sikkim. Mahamuni (2015) found that the *B. cenocepacia* strain VIMP01 solubilized phosphate and potassium and also produced IAA, which was similar to our isolate *B. cenocepacia* SRD.

Phosphorous is considered as the second-most key nutrient after nitrogen for plant growth, although less than 5% of the total soil phosphorous is found in the available form to plants (Otieno et al., 2015). Hence, the capability to solubilize the insoluble form of phosphate is one of the key features of PGP bacteria to boost plant nutrition through an escalation in phosphorous uptake by plants (Taurian et al., 2010). The application of these types of PSB in the soil might contribute to the reduction of excessive usage of the chemical fertilizers, and thereby, it improves the soil health of agricultural lands (Taurian et al., 2010).

In the present investigation, the three bacterial consortia (Consortia-1, Consortia-2, and Consortia-3) were prepared based on solubilization of phosphate and potassium, and nitrogen-fixing ability of the bacterial isolates. Application of bacterial consortia at the rice fields of Pakyong during August 2019 showed that there were significant differences in all the rice plant growth and yield parameters, except leaflets number per plant in the rice plants treated with three different bacterial consortia as compared with uninoculated control. All three consortia significantly improved grains per panicle, grain weight, and root length as compared with uninoculated control plants. But Consortia-3-inoculated rice plants showed higher plant biomass, grains per panicle, grain weight, root length, and dry root weight as compared with control plants and other consortia-inoculated plants. The collective PGP traits and nutrient solubilization properties observed in Consortia-3 were due to the bacterial mixture of *K.oryzendophytica* YMA7, *P. kribbensis* KSB, and *P*. *polymyxa* COW3. Our results are in agreement with studies by Di Benedetto et al. (2019) and Devi et al. (2020), who had showed the PGP properties of *Bacillus* spp., *Pseudomonas* spp., and *Kosakonia* spp. Panneerselvam et al. (2019) showed that *B. subtilis* strain BioCWB (570 μg ml^–1^) and *B. luciferensis* strain K2 (417.3 μg ml^–1^) produced higher amounts of phosphates. Similarly, in our findings, *P. polymyxa* strain COW3 and *Bacillus* sp. strain ARA produced higher phosphates, i.e., 580 and 579 μg ml^–1^, respectively. In general, the application of Consortia-3 among all the three consortia significantly increased plant growth parameters as compared with those of the uninoculated control plant. Many previous studies have proved that *Bacillus* spp. (*B. aryabhattai*, *B. megaterium*, *Bacillus polymyxa*),*Pseudomonas* spp. (*P. kribbensis*, *P. rhodesiae*), *Burkholderia* spp., and *Kosakonia* spp. possessed PGP attributes, enhanced plant growth, and increased yield in several agricultural and horticultural crops (Park et al., 2017; Devi et al., 2020; Castanheira et al., 2015).

The difficulties encountered by the native bio-inoculants might be distinctive all throughout the planet, as the various abiotic factors such as the edaphic, climatic, and geological conditions of the local environment vary remarkably (Ojuederie et al., 2019; Orozco-Mosqueda et al., 2021). Consequently, for quite a long time, it has been attempted to separate local strains that permit to work on the harvests of similar regions from which they were secluded, which would recommend superior productivity to practice their valuable activities when related with plants in similar sorts of agricultural soils. Consequently, more investigation is needed to relate abiotic angles with the useful properties of each native consortia. Reduction of inorganic farming practices shall also prevent agriculturists from exposure to harmful chemicals that might be toxic not only to the soil but also to human health (Wightwick et al., 2010).

Native PGPR strains might help in plant growth development through various mechanisms. Direct enhancement might be through the improved nutrient accessibility and its proficiency in uptake, by increasing the capacity to solubilize P, to fix N2, and to create siderophores and plant growth hormones, for example, IAA (Glick, 2012). Native PGPR strains are the natural flora of the soil, yet their number is not sufficient to rival different microbes set up in the rhizosphere. Accordingly, the implementation of consortia developed from the native PGPR strains is important to increase the local population of the target microorganism and to boost their helpful properties for plant yield. The utilization of local soil bacterial consortia has many advantages when administered into the plant rhizosphere, as there might be less competition among themselves for nutrient cycling. Furthermore, they are more impervious to the local ecological stress conditions particularly experienced under the anticipated climatic changes (Vimal et al., 2017).

Long-term organic farming/agricultural practices can directionally change the bounty of certain bacterial phyla. Yet there is no adequate comparison of soil bacterial taxa in light of long-term organic farming/agricultural vs. inorganic farming practices. Long-term organic farming might expand the availability of natural C to choose for certain microbial taxa levels that feed fundamentally on natural substrates and multiply significantly, bringing about the progressions in microbial local area structure and soil supplement status (Cederlund et al., 2014). As a result, specific microbial taxa abundances might be considerably expanded by long-term organic farming and also should show some level of associations with soil supplements. Besides, these taxa might show a possible beneficial impact on crop efficiency and agro-biological system stability (Francioli et al., 2016). Network analysis of the taxa, as estimated by next-generation sequencing, might help with interpreting the complex microbial communities and the role of the environmental standards governing the local area (Barberán et al., 2012; Banerjee et al., 2016). Ongoing analysis through high-throughput sequencing shall un-reveal the microbial variety and local area arrangement under long-term organic farming and inorganic farming (Lentendu et al., 2014; Calleja-Cervantes et al., 2015; Zhou et al., 2015; Chen et al., 2016; Ding et al., 2016; Francioli et al., 2016; Li F. et al., 2017). Notwithstanding, very few studies have been done about which microbial taxa are firmly affected by long-term organic and inorganic farming practices and how these taxa are connected to soil supplement boundaries.

Regarding organic and inorganic farming alone, the former practice ordinarily delivers lower crop yield (Seufert et al., 2012); however, the latter causes more ecological issues (Davidson, 2009). The integrated technique of periodic alteration between organic and inorganic farming is assessed as the best method to upgrade crop productivity and increment of soil organic matter (SOM) level (Wei et al., 2016). In the interim, consolidated organic treatment improves the production of soil invertase, urease, and antacid phosphatase, which are three average microbial exoenzymes engaged with C, N, and P mineralization (Li F. et al., 2017). All the more significantly, in contrast with inorganic farming, the organic treatment improved more measures of explicit bacterial taxa. These taxa are involved in the decay of complex natural matters and soil supplement changes and are accordingly advantageous for plant development by working on supplement accessibility. Subsequently, we need to analyze the bacterial diversity to comprehend the long-term organic farming against inorganic farming in Sikkim. In any case, an essential issue lies in the fact that Sikkim has banned inorganic cultivating practices since 2003, so to mirror inorganic farming in a greenhouse is the solitary choice for better relative investigation.

## Conclusion

This is the first-ever study of native consortia developed from the rice rhizosphere of organic farmlands of Sikkim, which are found to be effective as an NPK enhancer so as to help in plant growth promotion. Also, they have antifungal properties that serve as additional crop security against fungal pathogens. We have obtained efficient P-solubilizing, K-solubilizing, N_2_-fixing, IAA-producing, and antagonistic potential bacteria present among the native rice soil rhizosphere. These characteristics are considered as important PGP traits; and the bacterial consortia prepared from N-, P-, and K-producing bacteria have been found effective in improving the growth and N, P, and K contents of tested rice plants. Consortia-3 (*K. oryzendophytica* YMA7 +*P. kribbensis* KSB +*P*. *polymyxa* COW3) showed promising PGP traits such as phytohormone production and nutrient solubilization abilities. In the rice plant, in the soil inoculated with bacterial Consortia-3, the N/P/K content was N (17.33 g kg^–1^), P (4.83 g kg^–1^), and K (37.33 g kg^–1^) as observed against the control plant N/P/K (7.0, 2.16, and 24.66 g kg^–1^, respectively). Three bacterial consortia developed in this study were first tested in greenhouse pot experiments and later applied to the rice field. Based on the agronomic parameters, significant increases were observed in all the plant growth and yield parameters except leaf number per plant when compared with uninoculated rice plants. Consortia-3 significantly improved grains per panicle (110 grain numbers per panicle), grain weight in grams (27.8 g), and root length in cm (9.5 cm) as compared with uninoculated control rice plants. These bacterial consortia can be potential candidates for bio-intensive nutrient management in organic farming systems. Further studies should be focused on the detailed synergistic effect for the production of N, P, K and functional characterization of bacterial consortia for practical applications in the field.

## Data Availability Statement

The datasets presented in this study can be found in online repositories. The names of the repository/repositories and accession number(s) can be found in the article/Supplementary Material.

## Author Contributions

MS performed investigation, conceptualization, experiments, visualization, and writing–original draft. NB and LS performed editing of the manuscript, acquired funding and resources, and provided supervision. SD performed statistical and data analysis, visualization, writing, and subediting of the manuscript. All authors contributed to the article and approved the submitted version.

## Conflict of Interest

The authors declare that the research was conducted in the absence of any commercial or financial relationships that could be construed as a potential conflict of interest.

## Publisher’s Note

All claims expressed in this article are solely those of the authors and do not necessarily represent those of their affiliated organizations, or those of the publisher, the editors and the reviewers. Any product that may be evaluated in this article, or claim that may be made by its manufacturer, is not guaranteed or endorsed by the publisher.
