# Supplementary material for: Isolation, Characterization, and Evaluation of Native Rhizobacterial Consortia Developed From the Rhizosphere of Rice Grown in Organic State Sikkim, India, and Their Effect on Plant Growth
Source: Front Microbiol. 2021 Sep 6;12:713660. doi: 10.3389/fmicb.2021.713660 (PMC8450577; doi:10.3389/fmicb.2021.713660)
Supplement: Supplementary file 1 [file Table_1.DOCX]

Supplementary Material

| **ST1 \| Physicochemical analysis of rice rhizospheric soil of sampling sites** | | | | | |
| --- | --- | --- | --- | --- | --- |
| **Sample Name** | **pH** | **SOC (%)** | **Available N (kg ha^-1^)** | **Available (kg ha^-1^)** | **Available K (kg ha^-1^)** |
| **Melli (M)** | 6.8 | 1.1 | 238 | 19 | 25 |
| **Sajong (S)** | 6.7 | 1.3 | 235 | 18.1 | 22 |
| **Assam Lingzey (AL)** | 6.5 | 1 | 239 | 16.1 | 21 |
| **Mean Value**  **(M, S, and AL)** | **6.6** | **1.13** | **237.3** | **17.7** | **22.6** |

**ST2 | Morphological and biochemical characterization of various isolates; (+) indicates positive and**

**(−) indicates negative results.**

| - **Biochemical test Carbohydrate assimilation test** | | | | | | | | | | | | | | |
| --- | --- | --- | --- | --- | --- | --- | --- | --- | --- | --- | --- | --- | --- | --- |
| - **Isolates** | - **Gram stain** | - **Form** | - **Indole** | - **Methyl red** | - **Voges Proskauer’s** | - **Citrate utilization** | - **Glucose** | - **Adonitol** | - **Arabinose** | - **Lactose** | - **Sorbitol** | - **Mannitol** | - **Rhamnose** | - **Sucrose** |
| - **SRB** | - **-** | - **Rod** | **-** | - **+** | - **-** | **+** | - **-** | **-** | - **-** | **-** | **-** | **-** | **-** | - **-** |
| - **COW3** | - **+** | - **Rod** | - **-** | - **+** | - **+** | **+** | - **+** | - **-** | - **+** | - **+** | **-** | - **+** | **-** | - **+** |
| - **KSB** | - **-** | - **Rod** | **-** | - **+** | - **-** | **+** | - **-** | **-** | - **-** | **-** | **-** | **-** | **-** | - **-** |
| - **SRD** | - **-** | - **Rod** | **-** | - **-** | **-** | **+** | **+** | **-** | **+** | **-** | **-** | **-** | **-** | - **+** |
| - **PSB2** | - **+** | - **Rod** | **-** | - **+** | **-** | **+** | **+** | **-** | **+** | **-** | **-** | **-** | **-** | - **-** |
| - **YMA7** | - **-** | - **Rod** | **-** | - **+** | **-** | **+** | **+** | **-** | **+** | **-** | **-** | **-** | **-** | **+** |
| - **PSB1** | - **+** | - **Rod** | **-** | - **+** | **-** | **+** | **+** | **-** | **+** | **-** | **-** | **-** | **-** | **+** |
| - **ARA** | - **+** | - **Rod** | **-** | - **-** | **-** | - **-** | **+** | **-** | **+** | **-** | **-** | - **+** | **-** | **+** |

| **ST3 \| Antagonistic activity of potential isolates against *Pestalotiopsis* sp., *Curvularia eragrostidis*, and *Colletotrichum gloeosporioides* under dual culture plate method.** | | | | | | |
| --- | --- | --- | --- | --- | --- | --- |
| **Strain** | ***Curvularia eragrostidis*** | | ***Pestalotiopsis* sp.** | | ***Colletotrichum gloeosporioides*** | |
|  | **Mycelial growth diameter (cm)** | **Percentage of inhibition over control** | **Mycelial growth diameter (cm)** | **Percentage of inhibition over control** | **Mycelial growth diameter (cm)** | **Percentage of inhibition over control** |
| SRD | 2.0±0.10^e^ | 43.0±1.0 ^b^ | 2.4±0.36^e^ | 29.3±0.57^c^ | 2.1±0.23^e^ | 90.0±0.0^a^ |
| ARA | 4.9±0.36^c^ | 28.6±0.57^ef^ | 3.2±0.26^d^ | 30.3±0.57^b^ | 5.4±0.36^c^ | 26.3±0.57^d^ |
| COW3 | 6.1±0.15^b^ | 32.6±1.15^c^ | 4.2±0.20^c^ | 30.0±0.0^bc^ | 5.2±0.57^c^ | 35.3±0.57^c^ |
| PSB1 | 2.0±0.05 ^e^ | 25.3±0.57^g^ | 3.1±0.23^d^ | 24.3±0.57^e^ | 2.2±0.57 ^e^ | 24.6±0.57^g^ |
| PSB2 | 1.6±0.10 ^f^ | 29.6±0.57^de^ | 2.0±0.15 ^e^ | 16.6±0.57^f^ | 1.6±0.15 ^e^ | 29.3±0.57^e^ |
| YMA7 | 1.4±0.15 ^f^ | 50.3±0.57^a^ | 2.4 ±0.36^e^ | 25.6±0.57^d^ | 2.4±0.26 ^de^ | 50.3±0.57 ^b^ |
| SRB | 4.1±0.17^d^ | 27.6±0.57^f^ | 3.5±0.30^d^ | 30.0±0.0^bc^ | 6.4±0.10 ^b^ | 20.6±0.57^h^ |
| KSB | 4.1±0.15 ^d^ | 31.3±2.30^cd^ | 5.1±0.15^b^ | 31.6±0.57^a^ | 3.1±0.10 ^d^ | 34.0±1.00^c^ |
| Control | 8.1±0.10^a^ | - | 7.7±0.20^a^ | - | 8.1±0.10^d^ | - |
| LSD  (p≤ 0.05) | 0.37 | 1.73 | 0.45 | 0.82 | 0.82 | 0.82 |
| CV | 5.66 | 3.38 | 6.96 | 1.98 | 11.8 | 1.39 |

Values are means ± SE. a,b,c,d,e,f letters on the bars denote differences on the basis of a t-test (p < 0.05)

| **ST4 \| Antagonistic activity of potential isolates against *Pestalotiopsis* sp., *Curvularia eragrostidis*, and *Colletotrichum gloeosporioides* under broth assay.** | | | | | | |
| --- | --- | --- | --- | --- | --- | --- |
| **Strain** | ***Curvularia eragrostidis*** | | ***Pestalotiopsis* sp.** | | ***Colletotrichum gloeosporioides*** | |
|  | **Mycelial dry weight (g)** | **Percentage of inhibition over control** | **Mycelial dry weight (g)** | **Percentage of inhibition over control** | **Mycelial dry weight (g)** | **Percentage of inhibition over control** |
| SRD | 0.43± 0.02^b^ | 49.0±1.7 ^b^ | 0.53±0.02 ^b^ | 33.0±2.0^a^ | 0.12±0.01 ^b^ | 91.0±1.0^a^ |
| ARA | 0.69± 0.02^b^ | 30.6±1.1^ef^ | 0.57±0.03 ^b^ | 33.6±0.57^a^ | 0.76±0.01 ^b^ | 27.3±2.51d^e^ |
| COW3 | 0.63±0.02 ^b^ | 35.3±1.5^cd^ | 0.52±0.02 ^b^ | 32.6±2.51^ab^ | 0.54±0.00 ^b^ | 36.3±1.15^c^ |
| PSB1 | 0.74±0.04 ^b^ | 28.3±3.0^f^ | 0.73±0.03 ^b^ | 26.3±1.52^bc^ | 0.78±0.01 ^b^ | 25.33±2.51^e^ |
| PSB2 | 0.71±0.02 ^b^ | 32.0±4.5^de^ | 0.79±0.02 ^b^ | 19.0±7.0^d^ | 0.61±0.04 ^b^ | 30.0±4.04^d^ |
| YMA7 | 0.44±0.02 ^b^ | 56.0±2.0^a^ | 0.77±0.03 ^b^ | 27.3±2.5^bc^ | 0.45±0.02 ^b^ | 53.0±3.05 ^b^ |
| SRB | 0.71±0.02 ^b^ | 31.0±2.6^ef^ | 0.69±0.03 ^b^ | 31.6±3.7^abc^ | 0.79±0.02 ^b^ | 22.0±2.08^e^ |
| KSB | 0.53±0.02 ^b^ | 37.0±2.0^c^ | 0.62±0.02 ^b^ | 35.0±2.0^a^ | 0.53±0.03 ^b^ | 35.6±4.50^c^ |
| Control | 0.82±0.04 ^b^ | - | 0.80±1.5^a^ | - | 1±0.0^a^ | - |
| LSD  (p≤ 0.05) | 1.85 | 4.23 | 0.89 | 5.41 | 0.04 | 4.76 |
| CV | 10.89 | 7.36 | 4.72 | 11.84 | 3.76 | 7.71 |

Values are means ± SE. a,b,c,d,e,f letters on the bars denote differences on the basis of a t-test (p < 0.05)

| **ST5 \| Soil available N/P/K status of rice field soil before application of consortia** | | | | | |
| --- | --- | --- | --- | --- | --- |
| **Sample Name** | **pH** | **SOC (%)** | **Available N (kg ha^-1^)** | **Available P (kg ha^-1^)** | **Available K**  **(kg ha^-1^)** |
| **Soil sample 1** | 6.5 | 0.9 | 254 | 14 | 22 |
| **Soil sample 2** | 6.5 | 1.1 | 223 | 18 | 28 |
| **Soil sample 3** | 6.6 | 1.2 | 241 | 13 | 19 |
| **Mean Value** | 6.53 | 1.0 | 239.3 | 15 | 23 |

| **ST6 \| Soil available N/P/K status of rice field soil after application of consortia (initial treatment)** | | | | | |
| --- | --- | --- | --- | --- | --- |
| **Sample Name** | **pH** | **SOC (%)** | **Available N (kg ha^-1^)** | **Available P (kg ha^-1^)** | **Available K**  **(kg ha^-1^)** |
| **Soil sample 1** | 6.1 | 1.1 | 311 | 28.1 | 32 |
| **Soil sample 2** | 6.0 | 1.3 | 314 | 25.2 | 37 |
| **Soil sample 3** | 6.2 | 1.3 | 319 | 27.1 | 40 |
| Mean | 6.0 | 1.2 | 314.66 | 26.8 | 36.3 |

| **ST7 \| Determination of N/P/K uptake of rice plant after 60 days of consortia application (second treatment) in field based study** | | | |
| --- | --- | --- | --- |
| **Sample Name** | **N content in rice plant (g/kg)** | **P content in rice plant (g/kg))** | **K content in rice plant (g/kg)** |
| **Consortia-1** | 8.66±0.57^c^ | 2.86±0.32^b^ | 25.66±0.57^c^ |
| **Consortia- 2** | 14.66±0.57^b^ | 4.83±0.28^a^ | 33.66±1.52^b^ |
| **Consortia -3** | 17.33±1.15^a^ | 4.83±0.28^a^ | 37.33±0.57^a^ |
| **Control** | 7.0±1.0^d^ | 2.16±0.28^c^ | 24.66±0.57^c^ |
| **LSD** | **1.27** | **0.54** | **1.86** |

Values are means ± SE. abcd letters on the bars denote differences on the basis of a t-test (p < 0.05)

**Supplementary Figure SF1: Growth profile of the bacterial isolates based on temperature, pH and NaCl concentrations.**

**
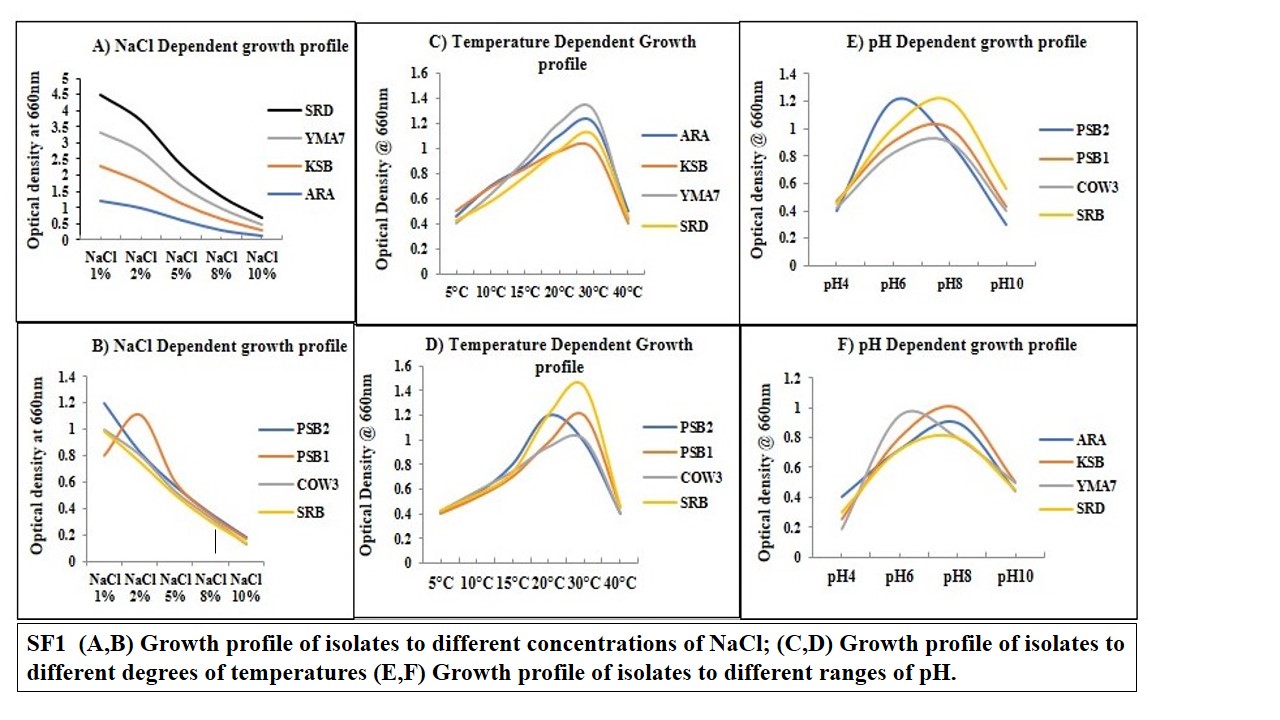
**

**Supplementary Figure SF2: Construction of the phylogenetic tree using the Maximum Likelihood method and Jukes-Cantor model.**

**
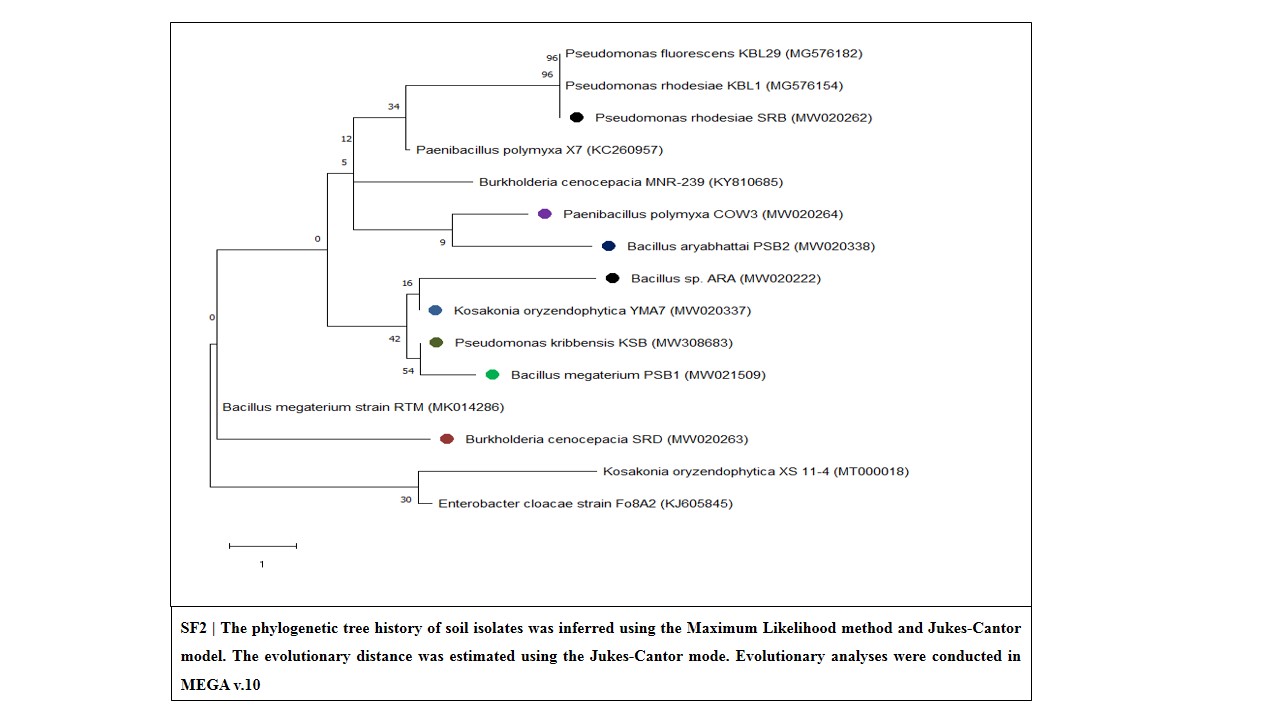
**

**Supplementary Figure SF3: Antifungal activity of the bacterial isolates.**

**
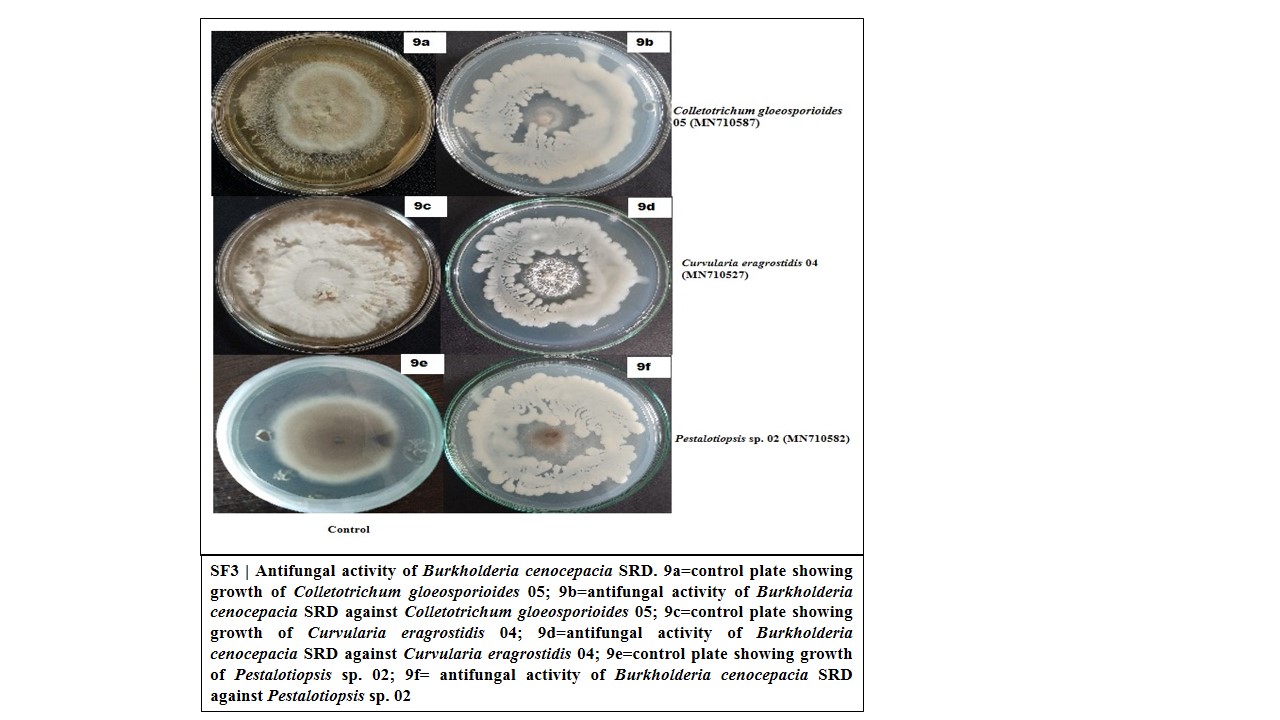
**

**Supplementary Figure SF4: Evaluation of rice grains post-treatment of consortia in rice field study.**


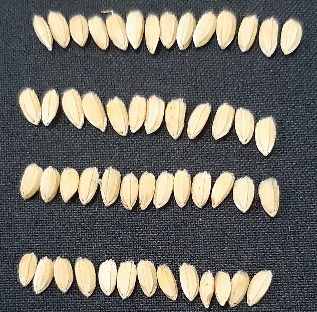

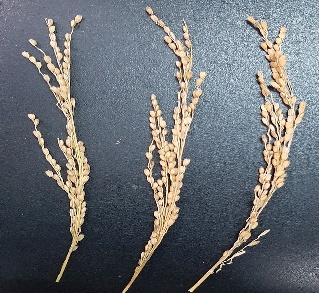

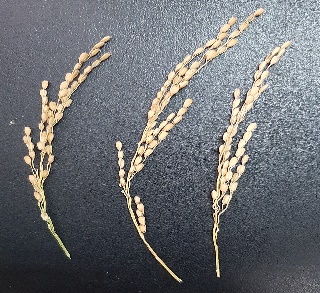

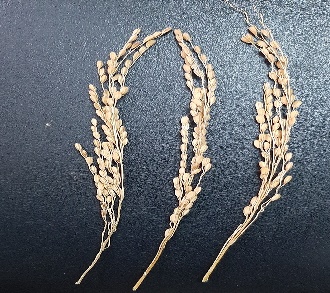

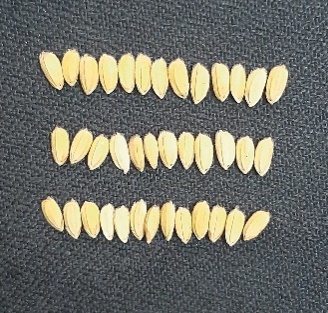

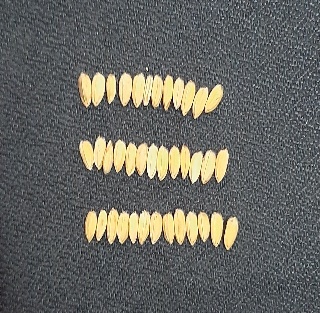


**A**

**C**

**D**

**E**

**SF4 | Evaluation of rice grains. (A, B) Consortia-1; (C, D) Consortia-2; (E, F) Consortia-3 and (G, H) Control rice plants at Pakyong field.**


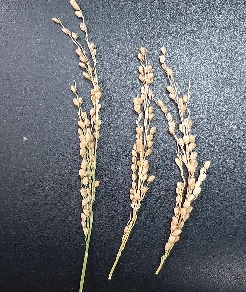


**G**


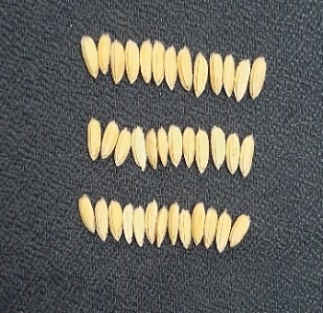


**B**

**F**

**H**

**Supplementary Figure SF5: Evaluation of rice grains post-treatment of consortia in rice field study.**


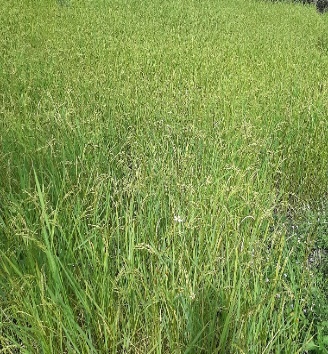

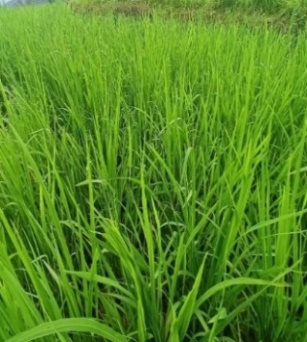

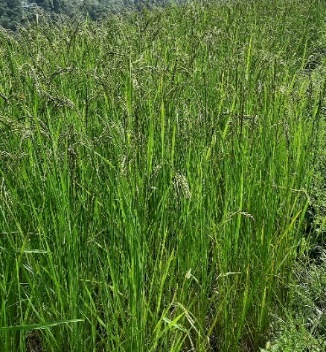

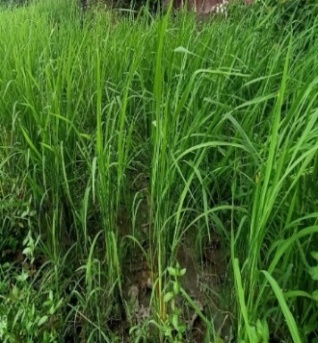

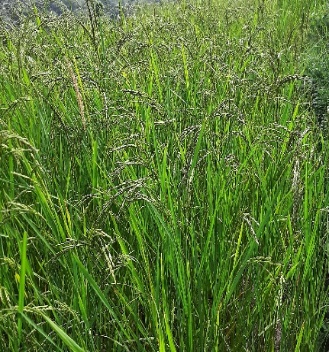

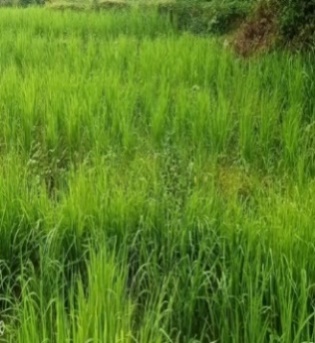


**A**


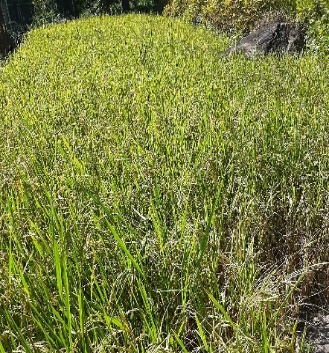

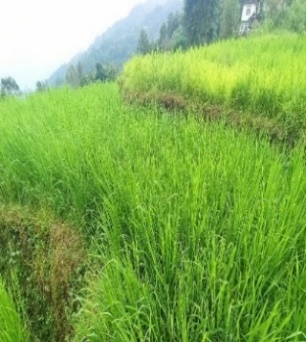


**SF5 | Field study. (A, B) Consortia-1; (C, D) Consortia-2; (E, F) Consortia-3 and (G, H) Control rice plants at Pakyong field.**

**C**

**F**

**B**

**D**

**E**

**G**

**H**
